# Depth of invasion (DOI) as a predictor of cervical nodal metastasis and local recurrence in early stage squamous cell carcinoma of oral tongue (ESSCOT)

**DOI:** 10.1371/journal.pone.0202632

**Published:** 2018-08-22

**Authors:** Muhammad Faisal, Muhammad Abu Bakar, Albash Sarwar, Mohammad Adeel, Fatima Batool, Kashif Iqbal Malik, Arif Jamshed, Raza Hussain

**Affiliations:** 1 Department of Surgical Oncology, Shaukat Khanum Memorial Cancer Hospital and Research Center, Lahore, Pakistan; 2 Department of Cancer Registry and Clinical Data Management, Shaukat Khanum Memorial Cancer Hospital and Research Center, Lahore, Pakistan; 3 Department of Radiation Oncology, Shaukat Khanum Memorial Cancer Hospital and Research Center, Lahore, Pakistan; Johns Hopkins University, UNITED STATES

## Abstract

**Background:**

The new AJCC staging system (8th edition) incorporates depth of invasion to stage oral cancers. It is a recognized predictor for neck nodal metastasis and local recurrence, the associated risk is not well defined. The aim of this study was to explore the risk of occult neck nodal metastasis and local recurrence in relation to depth in early stage squamous cell carcinoma of oral tongue.

**Methods:**

We have evaluated records of 179 patients with early tongue cancer treated in our unit from 2006–2015 with a mean age of 57.92 ± 11.93 years. Treatment modalities used were surgery (26%), surgery followed by radiotherapy (64%) and chemo-radiation (10%). Neck dissection was ipsilateral in 94% and bilateral in 6% of the patients. Patients were grouped according to the AJCC cut off points in 8^th^ edition for depth; group A: 1–5 mm (35%), group B: 6–10 mm (47%) and group C: > 10 mm (18%).

**Results:**

Risk of local recurrence and nodal metastasis for Group A was 15% (10/63) and 23% (15/63), group B 20% (17/84) and 34% (29/84), and group C 40% (13/32) and 53% (17/32).

**Conclusions:**

Depth more than 10 mm is associated with significantly increased risk of recurrence and nodal metastasis. Elective neck dissection should be a consideration for tumors having depth less than 5mm.

## Introduction

Head and neck cancer is common in several regions of the world and is on the rising trend in third world population [1]. Overall head and neck cancer accounts for more than 550,000 cases worldwide annually with a male to female ratio increasing from 2:1 to 4:1[2]. Almost 90% are squamous cell carcinoma and are the 6^th^ leading cause of death worldwide by incidence [3]. Tobacco, alcohol and recently Human Papilloma virus (HPV) are considered to be primary risk factors [4]. The most common intraoral site for head and neck cancer is the tongue, with squamous cell carcinoma (SCC) of the oral tongue being the predominant malignancy. Neck nodal metastasis has been established as the predictor of survival for oral cavity tumors. The risk of occult nodal metastasis is 27–40% for early stage squamous cell carcinoma of oral tongue (ESSCOT)[5]. Neck dissection is mandatory if the risk of nodal metastasis is more than 15–20% [6–9]. This poses more challenge to treat a relapsed neck owing to high incidence of extra capsular spread in patients treated with primary resection and leaving neck for “wait and watch”. Contrary to that, another school of thought believes that elective neck dissection done in these early stage cancers is more of an overtreatment. In current AJCC TNM classification (8^th^ edition), depth of invasion (DOI) has been incorporated into T staging and has shown to be an important factor in redefining the staging system resulting in up gradation based on depth of invasion cut off 5mm and 10 mm [10]. Pentenero et al. has defined depth of invasion to be the distance from the deepest level of invasion to the reconstructed mucosal surface [11].

The aim our study was to explore the risk of occult neck nodal metastasis and local recurrence in relation to depth of invasion in early stage squamous cell carcinoma of oral tongue.

## Materials and methods

We have retrospectively evaluated records of 179 patients treated in Head and Neck Oncology unit of Shaukat Khanum Memorial Cancer Hospital and Research Centre, Lahore, Pakistan from 2006–2015 who were included based on the criteria of histopathologically proven early tongue cancers (T1, T2) with clinically negative neck (cN0) treated with surgery alone to be followed by radiotherapy or chemo radiation therapy in adjuvant setting. Due to retrospective nature of the study, it was granted exemption from Institutional review board (IRB). Diagnostic work up for all patients involved clinical examination; imaging modalities usually magnetic resonance imaging and chest x-ray to evaluate regional and distant metastasis respectively.

## Treatment modality

Surgery was used as the primary treatment modality aiming clear resection margins (1cm). Elective neck dissection (END) was performed in all cases addressing level 1–3 and extended to involve level 4 only if suspicious nodes were found intra-operatively. Patients with pathologically involved nodes (pN+) or close margin (<5mm) underwent post-operative radiotherapy (PORT) while those with multiple involved levels or extra nodal extension (ENE) received chemo-radiotherapy (CRT). Only 3 patients with involved margin were managed by re-excision to be followed by post-operative radiotherapy (PORT).

## Measuring depth of infiltration

Proper gross techniques (avoidance of tangential cuts and serial sectioning of the lesion at 2–3 mm intervals) facilitated subsequent microscopic assessment.

While thickness and DOI are often regarded as synonymous, they have slight differences. Thickness is usually measured from the mucosal surface of the tumor to the deepest point of tissue invasion in a perpendicular fashion with an optical micrometer or transparent ruler overlaid on the slide, while DOI is measured from the basement membrane of adjacent normal to the deepest point of invasion of the tumor. Measurement was done by experienced pathologists in millimeters.

## Statistical analysis

Descriptive analysis was done using summary measures for categorical variables and well as continuous variables. For categorical variables percentages (proportion) were used and for continuous variables mean and standard deviation were reported. Bivariate analysis was done using chi-square test to establish the relationship between two categorical variables. For the continuous explanatory variables independent t-test was used to check the mean difference.

Univariable analysis was done using cox regression model. During univariable analyses, the association of one explanatory variable at a time with the outcome variable was tested. In the multivariable model, statistical analysis was done using step-wise regression technique. All factors with p<0.05 were considered significant independent risk factors affecting recurrence and nodal metastatic. Final model was built using the likelihood test. The Kaplan-Meier method was used to estimate survival as a function of time, and survival differences were analyzed by the log-rank test. Statistical significance was defined as a two-tailed p-value 0.05. Statistical analysis was carried out using the SPSS software (version 20.0; SPSS, Chicago, IL, USA).

## Results

Table 1 presents the baseline description of 179 squamous cell carcinoma patients with a mean age and standard deviation of 57.92 ± 11.93 years. The majority of the patients were male (57%) with around (45.81%) had cT2 stage. In addition, more than half of the patients had pT1 stage (63.69) with (34.08%) had pN1 stage. Moreover, most of the patients had well differentiated grade (54.75%). Additionally, 20% of the patients had perineural invasion and only (3.91%) developed extra-capsular spread. Furthermore, depth of invasion above 10 millimeter was established in (17.88%) and (34.08%) developed nodal metastasis with (22.35%) had recurrence. Association of clinicopathological characteristics with recurrence and nodal metastasis were presented in Table 2 and Table 3. The rate of occult metastasis in our series of early tongue tumors was 36.31% (29% for T1and 45% for T2 tumors). When incorporating depth of invasion, occult metastasis was 23% in tumors with DOI ≤5mm, 34% in tumors with DOI 6–10 mm and 53% in tumors with DOI > 10 mm. In our study model, Univariate analysis have also documented association of poor grading and T2 tumor size (sowing tumor burden/volume) with local recurrence (p = 0.08 and 0.09 respectively) as shown in Table 2. On the other hand, clinical tumor size stage (cT), pathological tumor size stage (pT), pathological nodal stage (pN), grade, perineural invasion, extra-capsular spread and depth of invasion were also statistically significant with nodal metastasis; hence there is an association as shown in Table 3.

**Table 1:** Baseline characteristics of 179 squamous cell carcinoma patients.

Variables Categories	Total = N (%)
**Age**	
- Mean ± SD*	57.92 ± 11.93
**Sex**	
- Male	(57%)
- Female	77 (43%)
**cT Stage**	
- cT1	54.19%)
- cT2	82 (45.81%)
**pT stage**	
- pT1	3.69%)
- pT2	65 (36.31%)
**pN stage**	
- pNO	5.92%)
- pN1	61 (34.08%)
**Grade**	
- Poorly differentiated	5.59%)
- Moderately differentiated	39.66%)
- Well differentiated	98 (54.75%)
**Surgical margins**	
- Negative	46.37%)
- Positive/Close	96 (53.63%)
**Perineural invasion**	
- No	9.89%)
- Yes	36 (20.11%)
**Lymphovascular invasion**	
- No	6.15%)
- Yes	168 (93.85%)
**Extracapsular spread**	
- No	6.09%)
- Yes	7 (3.91%)
**Depth of invasion**	
- Group A (up to 5mm**)	35.20%)
- Group B (6-10mm**)	46.93%)
- Group C (Above 10mm**)	32 (17.88%)
**Nodal metastasis**	
- No	5.92%)
- Yes	61 (34.08%)
**Recurrence**	
- No	7.65%)
- Local	9.50%)
- Regional	11.73%)
- Distant	2 (1.12%)

**cT =** Clinical tumor size; **pT =** Pathological tumor size; **pN =** Pathological nodal status

*standard deviation

**millimeter

**Table 2:** Association of clinicopathological characteristics with recurrence.

Variables Categories	Negative Recurrence 139 (77.65%)	Positive Recurrence 40 (22.35%)	p-value
**Age (years)**			0.31
- Mean ± SD*	58.06 ± 11.57	57.40 ± 13.24	
**Sex**			0.42
- Male	77 (75.49%)	24.51%)	
- Female	62 (80.52%)	15 (19.48%)	
**cT stage**			0.81
- cT1	76 (78.35%)	21.65%)	
- cT2	63 (76.83%)	19 (23.17%)	
**pT stage**			**0.09**
- pT1	93 (81.58%)	18.42%)	
- pT2	46 (70.77%)	19 (29.23%)	
**pN stage**			**0.001**
- pN0	102 (86.44%)	13.56%)	
- pN1	37 (60.66%)	24 (39.34%)	
**Grade**			**0.08**
- Poorly differentiated	5 (50.0%)	(50.0%)	
- Moderately differentiated	55 (77.46%)	22.54%)	
- Well differentiated	79 (80.61%)	19 (19.39%)	
**Surgical margins**			0.84
- Negative	65 (78.31%)	21.69%)	
- Positive/close	74 (77.08%)	22 (22.92%)	
**Perineural invasion**			0.19
- No	114 (79.72%)	20.28%)	
- Yes	25 (69.44%)	11 (30.56%)	
**Lymphovascular invasion**			1.00
- No	9 (81.82%)	(18.18%)	
- Yes	130 (77.38%)	38 (22.62%)	
**Extra-capsular spread**			0.19
- No	135 (78.49%)	21.51%)	
- Yes	4 (57.14%)	3 (42.86%)	
**Depth of invasion**			**0.02**
- Group A (up to 5mm**)	53 (84.13%)	15.87%)	
- Group B (6-10mm**)	67 (79.76%)	20.24%)	
- Group C (Above 10mm**)	19 (59.38%)	13 (40.63%)	

cT = Clinical tumor size; pT = Pathological tumor size; pN = Pathological nodal status

*standard deviation

**millimeter

**Table 3:** Association of clinicopathological characteristics with nodal metastasis.

Variables Categories	Nodal metastasis negative 118 (65.92%)	Nodal Metastasis positive 61 (34.08%)	p-value
**Age**			
- Mean ± SD*	57.69 ± 11.75	58.36 ± 12.35	0.64
**Sex**			0.13
- Male	72 (70.59%)	29.41%)	
- Female	46 (59.74%)	31 (40.26%)	
**cT stage**			**0.03**
- cT1	71 (73.20%)	26.80%)	
- cT2	47 (57.32%)	35 (42.68%)	
**pT stage**			**0.05**
- pT1	81 (71.05%)	28.95%)	
- pT2	37 (56.92%)	28 (43.08%)	
**pN stage**			**0.001**
- pN0	117 (99.15%)	(0.85%)	
- pN1	1 (1.64%)	60 (98.36%)	
**Grade**			**0.01**
- Poorly differentiated	5 (50.00%)	(50.00%)	
- Moderately differentiated	39 (54.93%)	45.07%)	
- Well differentiated	74 (75.51%)	24 (24.49%)	
**Surgical margins**			0.29
- Negative	58 (63.88%)	30.12%)	
- Positive/close	60 (62.50%)	36 (37.50%)	
**Perineural invasion**			**0.02**
- No	100 (69.93%)	30.07%)	
- Yes	18 (50.00%)	18 (50.00%)	
**Lymphovascular invasion**			0.19
- No	113 (67.26%)	32.74%)	
- Yes	5 (45.45%)	6 (54.55%)	
**Extra-capsular spread**			**0.01**
- No	117 (68.02%)	31.98%)	
- Yes	1 (14.29%)	6 (85.71%)	
**Depth of invasion**			**0.02**
- Group A (up to 5mm**)	48 (76.19%)	23.81%)	
- Group B (6-10mm**)	55 (65.48%)	34.52%)	
- Group C (Above 10mm**)	15 (46.88%)	17 (53.13%)	

cT = Clinical tumor size; pT = Pathological tumor size; pN = Pathological nodal status

*standard deviation

**millimeter

In multivariable analysis, two variables were identified as significant independent risk factor for recurrence: pN1 stage (adjusted odds ratio [AOR] 3.77; 95% confidence interval [CI] (1.98–7.20), p-value (0.01) and depth of invasion; group B (AOR 1.09; 95% CI (0.49–2.41), 0.83) and group C (AOR 2.99; 95% CI (1.30–6.88), 0.01) as shown in Table 4. Furthermore, independent risk factors for nodal metastasis; cT2 (AOR 2.18; 95% CI (1.25–3.82), 0.01), perineural invasion; (AOR 2.32; 95% CI (1.25–4.31), 0.01 and extra-capsular spread; (AOR 4.40; 95% CI (1.80–10.89), 0.001) as shown in Table 5.

**Table 4:** Risk factors of recurrence.

Variables Categories	Unadjusted OR (CI), p-value	Adjusted OR (CI), p-value
**pN stage**		
- pN0	**Ref**	**Ref**
- pN1	3.71 (1.97 6.99), 0.001	3.77 (1.98 7.20), 0.001
**Depth of invasion**		
- Group A (up to 5mm**)	**Ref**	**Ref**
- Group B (6-10mm**)	1.38 (0.63 3.03), 0.42	0.49 2.41), 0.83
- Group C (Above 10mm**)	3.29 (1.44 7.54), 0.01	2.99 (1.30 6.88), 0.01

pN = Pathological nodal status

**millimeter

**Table 5:** Independent risk factors of nodal metastasis.

Variables Categories	Unadjusted OR (CI), p-value	Adjusted OR (CI), p-value
**Depth of invasion**		
- Group A (up to 5mm**)	**Ref**	**Ref**
- Group B (6-10mm**)	1.56 (0.83 2.94), 0.16	0.52 2.02), 0.93
- Group C (Above 10mm**)	2.92 (1.46 5.86), 0.003	1.69 (0.80 3.58), 0.17
**cT stage**		
- cT1	**Ref**	**Ref**
- cT2	2.25 (1.33 3.81), 0.002	2.18 (1.25 3.82), 0.01
**Perineural invasion**		
- No	**Ref**	**Ref**
- Yes	2.84 (1.59 5.10), 0.001	2.32 (1.25 4.31), 0.01
**Extra-capsular spread**		
- No	**Ref**	**Ref**
- Yes	7.14 (2.93 17.36), 0.001	4.40 (1.80 10.89), 0.001

cT = Clinical tumor size

**millimeter

## Discussion

Tongue as a subsite of oral cavity has shown higher risk of cervical nodal metastasis as compared to other subsite probably as a result of abundant lymphatics of tongue and floor of the mouth (FOM) [12]. Almost 25% of patients with Oral tongue squamous cell carcinoma present with occult metastasis at presentation [13]. Neck dissection apart from glossectomy has been recommended only if the risk of metastasis is close to 15–20% [8]. Previously, staging was based on superficial evaluation of tumor extension but current AJCC, Cancer Staging Manual, 8^th^ edition has incorporated depth of invasion which by definition is the distance from the deepest level of invasion to the reconstructed mucosal surface. It has further been classified as less invasive ≤ 5mm, moderate invasive 6 to10mm to deeply invasive ≥ 10 mm. A lot of data has been published concerning relation of tumor thickness or depth of invasion to cervical nodal metastasis with many studies emphasizing its role as a valid predictor [8, 9, 14, 15, 16]. O-charoenrat et al. have used 5mm as cut off and demonstrated that 5-year survival was 95% with tumor thickness of 5mm and 30% when tumor thickness was >5 mm (P = 0.002) [17]. In our study, we have used cut off at 5mm and 10mm using the same categorization as recommended by AJCC, Cancer staging manual, 2017. Many studies have emphasized the depth of invasion as the only or the most relevant predictor of prognosis [18, 19]. Interestingly, the five year survival for recurrent tumors in group A and group B was 85% and 74% respectively while for group C, it has dropped to 45%. Balasubramanian et al. in 2014 have shown risk of nodal metastasis from anterior tongue and floor of mouth. The rate of nodal metastasis for tongue cancer of a thickness 2.1-4mm was only 11.2%. This increased to 38.5% in patients with tongue cancers that were 4.1–6 mm thick [20]. These authors advocated neck dissection for tumors >4 mm thick for tongue tumors. Fakih et al. proposed a neck dissection for patients with a tumor depth of more than 4 mm [21]. Weiss et al. suggested a neck dissection if the probability of occult cervical metastasis is greater than 20% [22]. With increasing depth of invasion particularly > 10mm, there is significantly increased risk of occult nodal metastasis (53%) and decrease in 5 year survival to 45% (Table 3) Surprisingly, group A patients with DOI < 5mm have also shown occult metastasis to be present in 23% individuals mandating elective neck dissection even in early stage superficial tongue tumors.

Previous studies were more focused in determining the role of depth of tumor to cervical metastasis but nothing has been documented in showing its role in local recurrence. Our data has demonstrated local failure rate to be 9.5%, regional metastasis in 11.7% and distant metastasis in 1.1% of the patients. Group A showed 15% local recurrence rate while for Group B and Group C, it is 20% and 40% respectively (p-value = 0.02). With depth more than 10 mm, the risk of local recurrence increase by almost 3 times. This has been possibly attributed more towards the deeper tumors resulting in perineural invasion and close deep margins. Perineural invasion was positive in 11 of 29 patients who developed local recurrence. In previous staging system, tumors with T1 stage but depth > 1cm were still considered stage 1 disease and spared adjuvant treatment. As per AJCC staging system 8^th^ edition, tumors with depth > 1cm irrespective of their T stage must receive adjuvant radiotherapy to minimize the risk of local recurrence.

Based on the earlier TNM classification without incorporating the depth of invasion, the rate of occult metastasis in pathological T1 tumors and T2 tumors was 28 and 43% respectively. When re-staged by including depth of invasion, the rate was 23% for group A (T1 tumors with depth < 5mm), 34% for group B (T2 tumors with depth 6-10mm) and 53%for group C (T3 tumors with depth > 10 mm). Only 10% (5/66) of patients with occult nodal metastasis were positive for extra-capsular spread. Multivariate analysis for association of tumor depth and nodal metastasis has shown that tumors with more than 10 mm depth were 3 times more at risk of developing occult nodal metastasis. From the treatment perspective, neck dissection now seems to be a mandatory component of treatment even for early stage superficial T1 tumors with depth less than 5 mm because of the associated 23% risk of occult nodal metastasis.

As far as the treatment modalities used are concerned, surgery only has resulted in 95.8% disease control rate in group A as compared to Group B and C which is 87.5% and 66.7% respectively. But interestingly, adding radiotherapy has resulted in control rates of 83%, 81% and 60% for Group I, II and III respectively.

The drawback of our study is its retrospective nature. More accurate measurement tool and standardized technique apart from prospective design of the study correlating imaging technique and histopathological measurements in determining tumor thickness are the next steps to follow.

## Conclusion

Our study has postulated that depth of invasion > 10 mm to be significantly associated with decreased 5 year survival and increased risk of occult metastasis. At the same time, depth < 5mm have significant risk of occult nodal metastasis (>20%) compelling elective neck dissection even in early stage oral squamous cell carcinoma of tongue.

## Supporting information

S1 Dataset Dataset of manuscript. (SAV)

## Abbreviations

SCC: Squamous cell carcinoma
HPV: Human Papilloma virus
ESSCOT: Early stage squamous cell carcinoma of oral tongue
DOI: Depth of invasion
END: Elective neck dissection
PORT: Post-operative radiotherapy
ENE: Extra-nodal extension
CRT: Chemo-radiotherapy
